# Genetic testing and genomic analysis: a debate on ethical, social and legal issues in the Arab world with a focus on Qatar

**DOI:** 10.1186/s12967-015-0720-9

**Published:** 2015-11-14

**Authors:** Hatem El Shanti, Lotfi Chouchane, Ramin Badii, Imed Eddine Gallouzi, Paolo Gasparini

**Affiliations:** Qatar Biomedical Research Institute, Hamad Bin Khalifa University (HBKU), Doha, Qatar; Laboratory of Genetic Medicine and Immunology, Weill Cornell Medicine-Qatar, Qatar Foundation, Education City, Doha, Qatar; Molecular Genetics Laboratory, Hamad Medical Corporation, Doha, Qatar; Hamad Bin Khalifa University (HBKU), College of Sciences and Engineering, Life Sciences Division, Doha, Qatar; Division of Experimental Genetics, Sidra Medical and Research Center, Doha, Qatar

**Keywords:** Genomic analysis, Genetic testing, Consanguinity, Islamic view, Genome projects

## Abstract

In 2013 both Saudi Arabia and Qatar launched genome projects with the aim of providing information for better diagnosis, treatment and prevention of diseases and, ultimately to realize personalized medicine by sequencing hundred thousands samples. These population based genome activities raise a series of relevant ethical, legal and social issues general, related to the specific population structure as well as to the Islamic perspective on genomic analysis and genetic testing. To contribute to the debate, the Authors after reviewing the existing literature and taking advantage of their professional experience in the field and in the geographic area, discuss and provide their opinions. In particular, the Authors focus on the impact of consanguinity on population structure and disease frequency in the Arab world, on genetic testing and genomic analysis (i.e. technical aspects, impact, etc.) and on their regulations. A comparison between the Islamic perspective and the ethical, social and legal issues raised in other population contexts is also carried. In conclusion, this opinion article with an up-to-date contribution to the discussion on the relevance and impact of genomic analysis and genetic testing in the Arab world, might help in producing specific national guidelines on genetic testing and genomic analysis and help accelerate the implementation and roll out of genome projects in Muslim countries and more specifically in Qatar, and other countries of the Gulf.

## Consanguinity, diseases and the Arab world

The history of Arabs extends more than 5000 years. Around 3500 BC, Semitic-speaking people of Arabian origin migrated into the valley of the Tigris and Euphrates rivers in Mesopotamia, eventually becoming the Assyro-Babylonians. About 2500 BC, another group of Semites left the Arabian Peninsula and settled along the eastern shores of the Mediterranean; some of these migrants became the Amorites and Canaanites of later times. Starting 7th century, Arabs, proclaiming the new religion of Islam, ventured from the Arabian Peninsula and conquered the wide area from the Persian/Arabian Gulf to the Atlantic Ocean [1, 2]. Today, the geographic area of the Arab world covers about 14 million km^2^ and spans two continents covering a distance of 6375 km. In 2004, the Arab population had an estimated size of more than 300 million people, contributing to 5 % of world populations, living in 22 countries across North Africa and West Asia, including the Middle East [1]. Residents share common demographic features, including a large family size, high rates of consanguinity, and rapid population growth [2, 3].

**Fig. 1:**
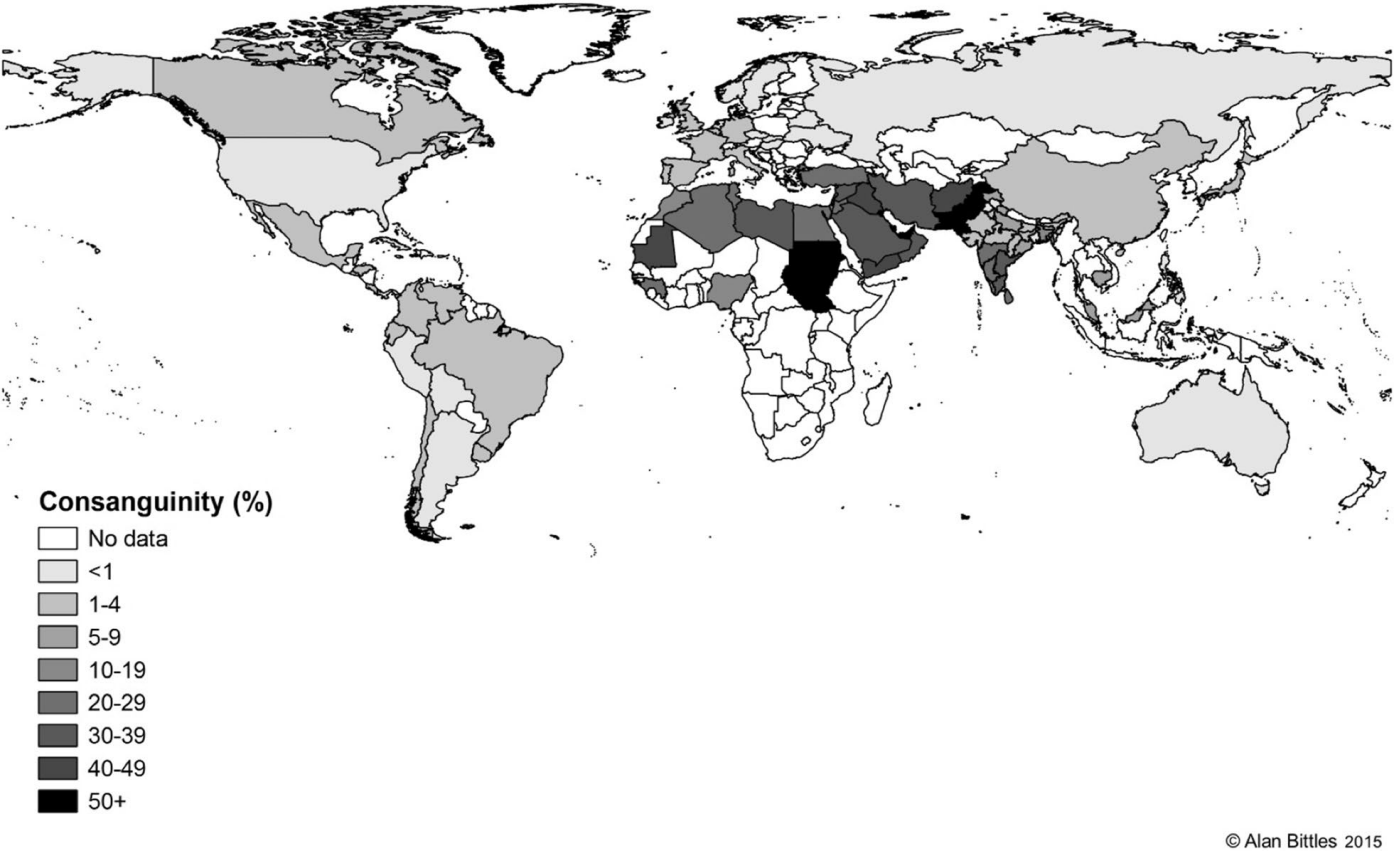
Global total consanguinity rates (extracted from [4])

Figure 1 shows that consanguinity is a prevailing tradition and is highly respected in most populations of North Africa, the Middle East and West Asia. First cousin unions are the most common, comprising 20–30 % of all marriages in given populations [4].

The rates and trends of first cousin marriages and other consanguineous marriages can vary hugely within and between populations and communities. Many factors determine these variances and they include ethnicity, religion, culture and geography. Emigrant communities from highly consanguineous countries such as Turkey, Pakistan and the Maghreb that are now resident in Europe, North America and Australia continue with the tradition of consanguineous marriages [4].

Consanguineous marriages are favored due to many sociocultural factors: the upholding of family groups and property, the better understanding of marital arrangements, the already established relationships with in-laws, and the monetary assets relating to dowry. Moreover, populations that favor consanguineous marriages believe that marrying within one’s family reduces health and financial risks [5]. In the Middle East, consanguinity is not restricted to just the Muslim communities. Other Middle Eastern religious groups, including the Lebanese, Jordanian, and Palestinian Christian communities, also perpetrate the tradition of consanguineous marriage, even though at a lesser rate than their Muslim counterparts and usually of lesser closeness [6–8].

Consanguineous marriages are highly regarded and are used to preserve wealth. They also present the ideal chance to maintain cultural stability and values [4, 5]. Consanguineous marriages are therefore believed to offer more stability for the married couple than marriages between non blood relatives. In most Arab societies, first cousin marriages are respected for many reasons. These marriages unite members of the same family as well as preserve the education of the offspring perpetrating the same family values. These views become even more significant in times of social changes and political or socioeconomic insecurity [9, 10].

Consanguineous marriage becomes possible when there is available close kin of comparable age with similar socioeconomic standing as well as a traditional system that advocates for or against distinct types of consanguineous marriage [11]. In the past, distant consanguinity was a rather familiar practice in rural communities. This custom eventually faded in Western societies as urbanization and industrialization in the nineteenth century increased. The two world wars of the twentieth century further contributed to the decline of consanguineous marriage in Western societies [12].

In the Middle East, some regions have witnessed a decline in their consanguinity rates such as Jordan, Lebanon and Palestine. Several factors could have contributed to this decline: a more educated female population, smaller families offering a decreased number of marriageable close kin, growing urbanization and improved family finances. As the rates of infectious diseases decline, more attention is given to the health effects of consanguineous marriages. This increased public health awareness could have also contributed to the decline in consanguineous marriage rates [6–8].

The decline in consanguineous marriage rates does not apply to all regions of the Middle East. Some Arab countries such as Qatar are seeing no significant reduction in their rates of consanguineous marriages. This could be attributed to the limited knowledge among the public regarding the nature of genetic risks that consanguineous marriages may present [4, 13, 14]. For these populations, the social benefits of close kin marriage outweigh any health risks. Society, culture, and politics still exercise a lot of influence in promoting intrafamilial marriages.

In populations where consanguinity and endogamy are practiced, the probability that a husband and wife are carriers (heterozygotes) for the same gene mutation that has been passed down to them from a common ancestor increases. Furthermore, this husband and wife couple will probably have many children. Consequently the odds that one or more of their children will inherit two copies of the ancestral recessive mutation, one from each parent, is increased [3, 5]. In populations with a high consanguinity rate, one also expects a higher rate of recessive genetic disorders. This correlation, however, will be less noticeable in urban communities, as family size tends to be smaller [5, 15, 16].

Some studies have shown a relationship between consanguinity and some genetic conditions and health problems such as phenylketonuria (PKU) [17], immunodeficiency disorders [18], children’s hypertension [19], beta-thalassemia [20], protein-C and protein-S deficiency [21] and low birth weight [22]. Al Bu Ali et al. found that first cousins marriages represented the most significant risk factor for birth defects and inborn errors of metabolism [23].

A few studies have reported a lack of effects of consanguinity or even a mild protective effect on the risk of developing diseases like breast cancer. For example, Arab women whose parents are consanguineous displayed reduced breast cancer risk compared to those women whose parents are non-consanguineous [24, 25]. This might be due to that the long-term practice of consanguinity may decrease the frequency of deleterious gene or eliminate it from a population.

It has been observed that no association exists between consanguineous marriage and Down’s syndrome. Nevertheless, it was noted that in some populations, the frequency of Down’s syndrome was elevated [26]. Zlotogora and Shaley observed that in an Arab village in Israel, cases of Down’s syndrome within the same endogamous group could not be explained by advanced maternal age alone [27].

The connection between consanguinity and major congenital malformations remains controversial [5]. It was shown that there was significantly increased risk of specific congenital heart defects in first cousin off-spring [28]. On the other hand, researchers conducted a genome-wide linkage analysis in South India, where uncle-niece and first cousin marriages are largely favored, and failed to determine a single gene of major effect in a clinically heterogeneous sample of consanguineous cases [29]. As regards to Qatar a survey published in 2010 [10] has shown that a vast majority of the population did not know that consanguinity had been linked to serious genetic diseases or that distant cousin marriages were genetically less risky than unions between first cousins. Moreover, genetic data linked to consanguinity are available for hearing loss. In this case, despite the presence of clear inbreeding and different levels of consanguinity a quite significant amount of genetic heterogeneity was detected [30].

### Final statement

The issue of consanguinity in Qatar and the Gulf region attracts much public attention. In many cases this issue is viewed negatively as a problem for both the family and the country. However, recent data clearly confirms that consanguinity has a minor impact on increasing disease frequency. Attention should be paid to:

1. Improving education about the health effects of consanguinity to help avoid feelings of guilt among the consanguineous populations.
2. Educating the physicians on the importance of focusing on some specific diseases whose frequency might be increased due to consanguinity, for example: thalassemia, hearing loss, intellectual disability, etc.
3. Offering premarital genetic screening for the most common population specific genetic diseases.

## Genetic testing: general principia and the Islamic perspective

The term “genetic testing” refers to a series of techniques including molecular analysis of human nucleic acids (DNA and RNA). Genetic tests are used as a health care tool to detect gene variants associated with a specific disease or condition and for studying complex and quantitative traits such cardiovascular diseases, cancer, diabetes, etc. Other fields include (a) pharmacogenetics where genetic tests are used to predict the effectiveness of therapeutics and guide their administration, (b) nutrigenetics and lifestyle where tests are used to asses diet response, lifestyle habits, physical activity predisposition, etc. (c) and other non-clinical uses (paternity testing, forensics, cosmetics, etc.).

Prenatal genetic tests are carried out on pregnant women to detect abnormalities/mutations in the fetus. Post-natal genetic tests are carried out after birth. Moreover, depending on their ultimate purpose, genetic tests can be divided into the following categories: (1) diagnostic, used to determine if the person is affected by a genetic disorder, (2) presymptomatic and predictive, when there is a family history of a genetic condition, undergoing genetic testing before symptoms actually appear may show if the person is at risk of developing that condition, (3) carrier testing, to detect the carrier status for recessive genetic diseases, such as thalassemia or cystic fibrosis, or if someone belongs to an ethnic group that has a high risk of a particular genetic disorder (e.g. Tay–Sachs disease in Jewish populations). The identification of a carrier status has relevant reproductive implications, (4) newborn screening, aimed at screening for certain genetic abnormalities that cause specific conditions such as PKU. In such cases, care and treatment can begin immediately following positive test results, (5) preimplantation testing, aimed, during an in vitro fertilization process, at diagnosing embryos for genetic defects in order to implant only those without them.

Depending on the type of test to be carried out different sources of DNA can be used, such as blood samples, cheek and buccal swabs, saliva, amniotic fluid cells, chorionic villus sampling, fetal cord blood cells, etc.

At the very early beginning, genetic tests were only available to detect chromosomal abnormalities (also called karyotyping). Then, with the development of the “Southern Protocol”, genetic testing also became available to investigate genes causing rare and inherited disorders like thalassemia or sickle cell anemia. The availability of PCR protocols led to a spread of tests that are able to detect even single point mutations in a very large number of genes underlying many inherited diseases.

Nevertheless, the positive detection rate of genetic testing varies largely from 10 to 80 % or more depending on several different limiting factors: clinical and genetic heterogeneity, accuracy and sensitivity of the technology used, etc. More recently, thanks to a series of technological improvements and new high-throughput protocols, the variety of genetic tests has greatly increased to also include multiple genes tests [31]. As a matter of fact, the use of Massively parallel sequencing (MPS) technologies and in particular of targeted-resequencing (TRS) has allowed to assess multiple genetic causes for many genetic diseases such as Fanconi anemia, Usher syndrome, retinopathy, hearing loss, cancer, ataxias, cardiomyopathies, etc. Moreover, the use of other high-throughput technologies such as SNPs arrays enables to detect small size insertions or deletions of genetic material (molecular karyotyping) as well as run of homozygosity. Molecular karyotyping is now considered as a first-line diagnostic test in comparison to traditional karyotype analysis in several areas of postnatal chromosome analysis.

Once a genetic test is carried out, the information should be accurately transmitted and explained to the patient and professional experts. Genetic test results may present various different situations, detailed below.

The identification of a genetic cause, in other words, a positive diagnostic genetic test, will help physicians determine the right treatment (i.e. genotype–phenotype correlations) as well as develop a management plan which will allow relatives to receive accurate risk assessments from a genetic counselor. The genetic counsellor might help the couple in making more informed family planning decisions. In the case of predictive tests where a positive test does not necessarily mean that someone will get that disorder, it would be possible to make lifestyle changes that may decrease the risk of developing a disease or decrease its impact.

A negative result means that a genetic alteration was not detected by the test. The absence of results can be explained by (1) the accuracy and sensitivity of the test which does not fully guarantee a positive outcome, and (2) the presence of large genetic heterogeneity hampering the mutation detection. Of course, if someone does not have the genetic alteration, this finding does not necessarily mean he or she will never get the disease. Moreover, in some cases, a genetic test may not be able to provide helpful information for the presence of DNA variants whose pathogenic role is doubtful. The use of TRS (and more broadly MPS) increases the possibility to detect such “doubtful” variants leading to increased uncertainty instead of clear-cut results. In these situations, follow-up testing may be necessary and such variants might be reinterpreted over time, as we learn more and potentially develop functional assays. This finding raises the question of whether there is a legal or moral responsibility to report such variants and eventually to recall, in the future, patients and their relatives and if so, with whom does the responsibility lie?

In many societies subjects deal with the cultural fear of being associated with a genetic disease and the following possible stigmatization within the community. This finding might lead to social disadvantages such as (a) marriage refusal if subjects belong to families with a known genetic disease and (b) consideration of another marriage in presence of affected fetuses or children. All family members are affected by the negative connotations associated with the presence of a genetic disease especially female family members. In the absence of major and effective therapeutical protocols for inherited diseases, genetic testing can help in making earlier and presymptomatic diagnosis as well as preventing a genetic disease through a selective termination of an affected fetus, which, of course, raises continuous ethical debates in all societies including the Islamic ones. The Islamic schools of thought have agreed that abortion is possible and lawful if continuation of the pregnancy will endanger a mother’s life [32, 33]. Moreover, the Islamic Jurisprudence Council of the Islamic World League released in 1990 a religious advise (fatwa) allowing abortion in the presence of severe fetal malformations not amenable to treatment or leading to poor quality of life of patients and their relatives [34, 35]. Abortion should be carried out prior to ensoulment thus, as largely agreed upon in the Islamic world, is before 120 days post fertilization. Interestingly, Islamic religious rules can be used by Muslims worldwide as they are not restricted to the country where subjects were born or are resident [33]. Within this framework and despite the absence of established networks of referral centers for genetic testing and counseling in the Arab world, a series of pilot studies and experiences has been reported in all fields of genetic testing over the past decade. This has been made possible, thanks to the presence of certain research centers, international collaborations, and the increase in the availability of advanced DNA technologies for the molecular analysis. As regards to diagnostic tests for genetic diseases there is a quite long standing experience in providing molecular diagnosis for some common inherited diseases such as thalassemia, sickle cell anemia, cystic fibrosis, hearing loss, etc. As regards complex traits, recently, a large survey on Saudi women has demonstrated high interest in genetic testing for breast cancer [36]. An even larger experience has been obtained on prenatal diagnosis (PND), preimplantation diagnosis (PGD) and premarital screening for inherited disease. As regards PGD it resulted that this option is, in principle, acceptable in Saudi Arabia and might be considered for a range of different conditions [37, 38] with an accurate selection of couples that should be counseled appropriately. Interestingly, 86 % of couples that already have experienced thalassemia (i.e. one affected child) expressed interest in PGD and were well aware about the possible benefits of this technology to avoid another affected child [39]. Usually, also PND is widely accepted even if data are mainly available on thalassemia, one of the most frequent inherited diseases in these populations. Interesting examples on a very good PND acceptability have been reported for thalassemia in West Bank and Gaza, where all couples with affected fetuses opted for abortion [40], always for thalassemia in Saudi Arabia [34], and for monogenic disorders in Israeli Arabs [27]. Finally, as regards to premarital screening for carrier identification, usually a positive attitude towards this approach has been reported in Saudi Arabia for hemoglobinopathies [41], in Syria among university students [42], in Iran [43] and in Dubai [44]. Since the end of 2009, Qatar has started mandatory premarital genetic screening to fight against the population’s most prevalent genetic diseases, such as homocystinuria, cystic fibrosis and spinal muscular atrophy, with further optional tests for beta-thalassemia, sickle cell anemia, fragile-X among others. Currently, genetic tests for 27 most prevalent genetic diseases of Qatari population are provided by the Molecular Genetics Laboratory and genetic counselling is offered by the Clinical Genetics Department of Hamad Medical Corporation. Additional rarer tests are carried out by private companies.

### Final statements

1. Genetic risks should be accurately explained during pre- and post-genetic test counseling, preventive options including PND should also be presented and discussed and a formal discussion about termination of pregnancy and the Islamic fatwa should take place.
2. Improved educational plans should be developed to help improve the understanding of genetics and genetic-related diseases among the general population as well as the physicians.
3. Where applicable networks of referral centers for genetic testing should be established or better improved to take care of the large number of genetic diseases present in the region.

## Genetic testing in the new era (genomic analysis)

Since the completion of the sequencing of the human genome, the demand for genetic analysis in the human health care system has increased, and molecular genetic diagnostics are urgently needed. However, many genetic diseases are molecularly and clinically heterogeneous, and until recently, the available techniques lacked the necessary ability to analyze several genes in parallel. The recently introduced MPS technology of next-generation sequencing now offers the unique opportunity to extend molecular genetic analysis by introducing this high-throughput technique, and by developing tailor-made medical re-sequencing approaches for gene identification and for the diagnosis of heterogeneous disorders. Whole genome sequencing (WGS) allows for the identification of all the possible variants at once, including chromosomal copy number variants. Protein-coding genes make up 1 % of the human genome but contain about 85 % of disease-causing mutations [45]. Therefore, efficient strategies for selectively sequencing all coding regions of the genome (i.e. ‘‘Whole Exome Sequencing” or “WES’’) or a panel of genes (i.e. “Targeted Re-Sequencing” or “TRS”) have the potential to contribute to the understanding of rare and common human diseases [45, 46]. Recent data demonstrate the ability to capture more than 95 % of the targeted coding sequences with high sensitivity and specificity for the detection of both homozygous and heterozygous alleles. For dominant traits, we expect that many, whose causes have not yet been identified, will be explained by alleles that have been difficult to map by linkage analysis due to the lack of availability of large families. Possible explanations are: reduced penetrance, the presence of substantial locus heterogeneity, the presence of alleles that impair reproductive fitness to the extent that many affected subjects harbor de novo mutations. The finding of independent de novo mutations within the same gene among different unrelated cases would constitute compelling evidence of disease causation. Moreover, in the presence of locus heterogeneity, identification of a significant excess in the number of independent mutations in the same gene, versus that expected by chance, will constitute evidence that a disease gene has been identified. Mapping data, which defines the location of the disease locus, animal models that produce similar phenotypes, and compelling functional biology assays can all contribute to the identification of such loci. For recessive traits, affected subjects arising from consanguineous unions contain substantial mapping information (i.e. the disease locus is expected to be homozygous). Such evidence reduces the region that must be analyzed by bioinformatics analysis upon MPS. The clinical utility of WES is well documented and has implications for disease gene discovery and clinical diagnosis [47]. Moreover, given the current rate of scientific advancement in genomics, MPS costs will continue to decrease making this technology accessible to an ever widening audience. In this light, MPS will be also used to study and diagnose quantitative and complex traits, for screening purposes, as well as to simply satisfy people’s curiosity about what their genome can tell about them [48, 49] (http://www.personalgenomes.org).

However, all these technological and diagnostic achievements have not yet led to updated and comprehensive guidelines and algorithms for the genetic diagnosis of many genetic diseases, which may be easily accessed by all the stakeholders involved: patients, clinicians, and geneticists. For example, since the publication of the “Genetic evaluation guidelines for the etiologic diagnosis of congenital hearing loss” by the American College of Medical Genetics [49], only sparse guidelines have been described in relation to multi-gene screening approaches, some of them issued by hospital laboratories (http://www.cincinnatichildrens.org/service/g/genetic-hearing-loss/default/).

As to Qatar, the recent launch of the Qatar Genome Project will “chart a road map for future treatment through personalized medicine” in the country. The ambitious goal of providing each citizen with his/her own genome, will lead to the discovery of almost all disease genes affecting the Qatari population, as well as a better understanding of the interaction between genes and the environment. These findings will translate into more accurate and personalized treatments as well as preventive plans and new lifestyle indications. Moreover, this project will pioneer the use of new genomic tools in consanguineous populations hopefully showing the great potential that these new tools have to reshape healthcare delivery in Qatar, and in a more broad sense, in other regional countries and inbred populations.

Finally, beyond the rapidly evolving technical aspects, several key ethical issues arise in the clinical translation of MPS. In particular, the following issues should be taken into account:(a) Return of results, and in particular disclosure of ‘incidental findings’ (if such a term may still be used to refer to something we are searching for: (a) Green RC & Genomics [50]; (b) structuring the informed consent process and allowing decisions related to the return of results. Moreover, consents should be deeply focused on the relevance of privacy (see genomic privacy below), (c) special situations with relatives and children, including ‘duty to warn’ at risk relatives and family communication issues including the role of consanguinity and (d) genomic privacy. Genomes not only uniquely and irrevocably identify their owner but also will have privacy repercussions for any relatives because they potentially reveal half of the genome of the parents and children and a substantial fraction of that of siblings Despite the fact that we are living in a world characterized by an impressive erosion of individual privacy (web pages and social networks that disclose any kind of personal information, the possibility of confidential emails being widely circulated, etc.) it should be clear that genome analysis undercuts privacy to a new degree.

### Final statements

1. The use of MPS technologies (i.e. WGS, WES and TRS) to answer specific clinical testing indications might lead, under some specific circumstances, to results beyond the scope of the test itself (called ‘incidental’ or ‘secondary’ findings). Thus, stakeholders must agree and determine which findings should be disclosed, when and in what manner. A good example is the list of recommendations published in 2013 by the American College of Medical Genetics and Genomics. They include a series of conditions and genes that should be considered, on the basis of clinical outcome and therapeutical options, ‘mandatory’ to return.
2. The presence of variants with uncertain functional role raises a series of open questions. Have the subjects/patients the ‘right’ to receive all of their genetic information or should they only be informed of data that are currently considered clinically relevant data? In any case, what about reinterpretation? Who is going to provide it and when, how to further report the results, etc.? Again stakeholders should agree in advance on what actions to take.
3. So far, classical and standard models of informed consent for genetic testing have been widely used. They are mainly administered through a genetic counseling session carried out by clinical geneticists and/or genetic counselors. During the counseling, testing options, risks, benefits and limitations of genetic testing are discussed in detail. This approach is not feasible with MPS data because of the large amount of genomic information that is generated by MPS technologies. If we are to uphold the necessity to discuss thoroughly all the information obtained, then the process will be long and tiresome. One way of resolving this problem is to create very well trained and multidisciplinary teams which would include geneticists, physicians and bioinformaticians amongst others. In all cases, the consent form should be prepared accordingly and must reflect this broad and complex situation. Alternatively, an individual undergoing MPS may be requested to complete a short online course explaining the advantages and disadvantages of this technology.
4. Reiterating earlier discussion, genomic analysis may generate a wealth of new genetic information with clinical relevance. Some of this genetic data may also impact the patient’s relatives. For example, MPS data may identify a predictive risk factor for cardiovascular disease without any relevance to the patient: the patient’s biochemical and clinical parameters are normal, the patient leads a healthy lifestyle, etc. However, this data may be of possible value to other relatives who might have completely different lifestyle habits and additional genetic and or environmental risk factors. This finding is even more relevant in inbred populations such as the Qatari population where kinship is very high and genetic results from one individual can be used, at least in part, to draw conclusions related to his/her relatives.
5. Huge efforts should be made related to informing and educating the public as to the great potential benefits arising from data, but also on the possible negative repercussions for the individual and his/her family should the data be casually and widely shared.
6. Most likely, in the near future, genomic analysis will oblige the society to reevaluate the current standards of medical confidentiality and privacy, a situation similar to the extensive use of internet and how that has changed our perceptions of personal space and privacy. Henceforth, substantial resources should be allocated to rapidly develop appropriate policies, guidelines and rules.

## Regulations and legislations of genetic testing worldwide including the Arab world

In the USA, three different federal agencies play a role in the regulation of genetic tests: Centers for Medicare and Medicaid Services (CMS), Food and Drug Administration (FDA), and the Federal Trade Commission (FTC). The CMS is responsible for regulating all clinical laboratories performing genetic testing, ensuring their compliance with the Clinical Laboratory Improvement Amendments of 1988. The FDA has the largest authority in terms of regulating the safety and effectiveness of genetic tests. Finally, the FTC aims at regulating genetic test advertising to ensure that it is not false or misleading. Regulations on genetic testing were and are still provided by a series of reports and acts such as: (1) NIH-Department of energy task force on genetic testing recommendation in 1997; (2) secretary’s advisory committee on genetic testing report in 2000; (3) secretary’s advisory committee on genetics, health and society report in 2008; (4) the laboratory test improvement act in 2006; (5) genomics and personalized medicine act in 2007; (6) genetic information nondiscrimination act in 2008; (7) framework for regulatory oversight of laboratory developed test (LDTs) in 2014; (8) FDA notification and medical device reporting for LDTs always in 2014.

In the EU there is a lack of specific genetic legislation. Nevertheless, some general principles are clearly defined: genetic data pertaining to health is ‘sensitive data’ under the EU data protection directive, and is thus to be treated confidentially. Likewise, discrimination based on genetic features is prohibited in the EU member states. Despite this legislative deficiency at EU level, several national legislations have been approved so far. The first explicit law including genetic testing was enacted in France in 1994 (Loi 94–653 and 94–654, later revised in 2004 and 2011). Soon after, specific legislations have been approved in Norway, Spain, Italy, Germany, Austria, Portugal, Sweden and Switzerland. In any case, most countries have applicable provisions for genetic testing in the general legal framework governing other medical, laboratory and professional activities in biomedicine, and other legislation, such as in vitro fertilization and PGD, data protection, patient rights, and discrimination.

In Australia the provision of pathology services is regulated by the department of health and aging administration of the funding schemes for public health is by Medicare. Under the provisions of the health insurance act 1973 (Cth) (HI Act), only accredited laboratories are eligible for Medicare benefits for only those tests or services listed on the Medicare benefits schedule. Accreditation of testing services is administered by the national association of testing authorities (NATA), the only national accreditation body endorsed by the Commonwealth government to assess laboratory competency. The accreditation scheme is based on guidelines issued by the national pathology accreditation advisory council (NPAAC). Before providing a genetic test through the Medicare benefits it should be assessed in compliance with the NPAAC guidelines. Genetic testing services generally comply with, but are not legally bound to the ethical aspects of human genetic testing information paper and guidelines on genetic research released by the national health and medication research council (NHMRC).

China’s regulatory position on genetic tests was quite unclear until March 2014 when the China food and drug administration (CFDA) acted to impose regulatory constraints on medical institutions (and other providers) related to the provision of clinical genetic tests. As a first measure CFDA issued a ban on all medical applications of ‘gene sequencing technology products’ such as disease prevention (including prenatal diagnosis) and diagnosis, treatment and monitoring, health status assessment and genetic risk factor prediction. It applies to manufacture, import, sale or use (including software) of MPS technology and genetic testing.

According to a quite recent survey from UNESCO (UNESCO Cairo Office, 2011), only Lebanon has written a law specifically formulated to regulate genetic testing (No. 625 of 2004). For all the remaining Arab countries, the issue of the regulation of genetic testing has been only partially addressed. In particular, many Arab countries, Bahrain, Tunisia, UAE, Saudi Arabia, Palestine, Oman, Kuwait, Algeria, Egypt and Jordan, have referred to genetic testing in relation to other procedures such as premarital screening, reproductive medicine, forensic medicine, biobanking, etc. or directly to worldwide accepted international guidelines and regulations. In some other cases, such as Syria and Morocco several laboratories perform genetic testing despite the absence of specific legislation. As regards to Qatar, quite recently the Shafallah Genetics Medical Centre has produced a formal operation manual that includes guidelines on genetic testing and release of information, stating that genetic data should not be given to insurance companies, employers, schools, or governments, except after obtaining the full informed consent of the person tested. Prenatal diagnosis should be offered to those who need it, but without applying any pressure on parents to agree to such testing. Strong attention should be paid in providing protection to minors and disabled persons. Genetic counselling should be given in a compassionate and professional manner, offering guidance and allowing individuals and families to make an informed choice.

Till present, the Department of Biomedical Research of the Supreme Council of Health in Qatar has prepared several policies and guidelines related to genetic testing, however, these guidelines are only present in a specific policy on diabetes mellitus.

A summary of the web links for some of the above mentioned agencies is given in Table 1.

**Table 1:** Links of a series of regulatory agencies/commissions involved in regulations of genetic testing in different countries

Centers for Medicare and Medicaid services (CMS)	https://www.cms.gov/	USA
Food and Drug Administration (FDA)	http://www.fda.gov/	USA
Federal Trade Commission (FTC)	https://www.ftc.gov/	USA
Department of Health	http://www.health.gov.au/	Australia
NATA	http://www.nata.com.au/nata/	Australia
National Pathology Accreditation Advisory Council (NPAAC)	http://www.health.gov.au/npaac	Australia
National Health and Medication Research Council (NHMRC)	https://www.nhmrc.gov.au/	Australia
CFDA	http://eng.cfda.gov.cn/WS03/CL0755/	China
SCH	http://www.sch.gov.qa/home-en	Qatar
European Commission	http://ec.europa.eu/	Europe

### Final statements

1. The absence of clearly defined policies and regulations related to “sensitive data”, discrimination, data protection, and privacy in Qatar means that each genetic test provider center must develop its own policies based on international regulations governing this type of testing. In that effect, this current draft may be used as a helpful review.
2. Qatari academic and research centers together with the other major players in Qatar, should collaborate to work on producing specific national guidelines on genetic testing and genomic analysis to be approved and released by the department of biomedical research in the supreme council of health. This legal framework will help accelerate the implementation and roll out of the Qatar genome project.

## Conclusion

This opinion article provides an up-to-date contribution to the discussion on the relevance and impact of increasing knowledge on genomic analysis and genetic testing in the Arab world. This new data can potentially form a new resource from which scientists and physicians can bring fresh insights to the world genomics community with a strong impact on understanding the genetic bases of mendelian as well as complex diseases (i.e. cardiovascular diseases, diabetes, metabolic syndrome, etc.). In this light, this paper might help in producing specific national guidelines on genetic testing and genomic analysis and further help accelerating the implementation and roll out of genome projects in Muslim countries and more specifically in Qatar, and other Arab countries of the Gulf.

## References

[1] Davison R. Where is the Middle East? In: Foreign Affairs; 1960. p. 665–75.

[2] Teebi AS, Teebi SA (2005). Genetic diversity among the Arabs. Community Genet.

[3] Al-Owain M, Al-Zaidan H, Al-Hassnan Z (2012). Map of autosomal recessive genetic disorders in Saudi Arabia: concepts and future directions. Am J Med Genet A.

[4] In: Bittles A, editor. Consanguineous marriages in Arab societies. Consang Retrieved; 2015.

[5] Bittles A (2001). Consanguinity and its relevance to clinical genetics. Clin Genet.

[6] Khoury SA, Massad D (1992). Consanguineous marriage in Jordan. Am J Med Genet.

[7] Khlat M (1988). Consanguineous marriages in Beirut: time trends, spatial distribution. Soc Biol.

[8] Vardi-Saliternik R, Friedlander Y, Cohen T (2002). Consanguinity in a population sample of Israeli Muslim Arabs, Christian Arabs and Druze. Ann Hum Biol.

[9] Assaf S, Khawaja M (2009). Consanguinity trends and correlates in the Palestinian Territories. J Biosoc Sci.

[10] Sandridge AL, Takeddin J, Al-Kaabi E, Frances Y (2010). Consanguinity in Qatar: knowledge, attitude and practice in a population born between 1946 and 1991. J Biosoc Sci.

[11] Cavalli-Sforza L, Moroni A, Zei G (2004). Consanguinity, inbreeding and genetic drift in Italy monographs in population biology.

[12] Liu F, Elefante S, van Duijn CM, Aulchenko YS (2006). Ignoring distant genealogic loops leads to false-positives in homozygosity mapping. Ann Hum Genet.

[13] Bittles AH, Mason WM, Greene J, Rao NA (1991). Reproductive behavior and health in consanguineous marriages. Science.

[14] Othman H, Saadat M (2009). Prevalence of consanguineous marriages in Syria. J Biosoc Sci.

[15] Bittles AH (2002). Endogamy, consanguinity and community genetics. J Genet.

[16] El-Najjar M. Y (1996). Consanguinity in Kuwait. Coll Antropol.

[17] Teebi AS, Al-Awadi SA, Farag TI, Naguib KK, el-Khalifa MY (1987). Phenylketonuria in Kuwait and Arab countries. Eur J Pediatr.

[18] White AG, Raju KT, Abouna GM (1988). A six year experience with recurrent infection and immunodeficiency in children in Kuwait. J Clin Lab Immunol.

[19] Saleh EA, Mahfouz AA, Tayel KY, Naguib MK, Bin-al-Shaikh NM (2000). Hypertension and its determinants among primary-school children in Kuwait: an epidemiological study. East Mediterr Health J.

[20] al-Fuzae L, Aboolbacker KC, al-Saleh Q (1998). beta-Thalassaemia major in Kuwait. J Trop Pediatr.

[21] Mohanty D, Das KC, al-Hussain H, Naglen P, Eklof B, Marouf R (1996). Thrombophilia in ethnic Arabs in Kuwait. Ann Hematol.

[22] Al-Awadi F, Amin EK (1992). Factors affecting birth weight in Kuwait. Part II: pregnancy characteristics and health factors. J Egypt Public Health Assoc.

[23] Al Bu Ali WH, Balaha MH, Al Moghannum MS, Hashim I (2011). Risk factors and birth prevalence of birth defects and inborn errors of metabolism in Al Ahsa, Saudi Arabia. Pan Afr Med J.

[24] Bener A, Ayoubi HR, Ali AI, Al-Kubaisi A, Al-Sulaiti H (2010). Does consanguinity lead to decreased incidence of breast cancer?. Cancer Epidemiol..

[25] Denic S, Bener A, Sabri S, Khatib F, Milenkovic J (2005). Parental consanguinity and risk of breast cancer: a population-based case–control study. Med Sci Monit.

[26] Alfi OS, Chang R, Azen SP (1980). Evidence for genetic control of nondisjunction in man. Am J Hum Genet.

[27] Zlotogora J, Shalev SA (2010). The consequences of consanguinity on the rates of malformations and major medical conditions at birth and in early childhood in inbred populations. Am J Med Genet A.

[28] Yunis K, Mumtaz G, Bitar F, Chamseddine F, Kassar M, Rashkidi J (2006). Consanguineous marriage and congenital heart defects: a case–control study in the neonatal period. Am J Med Genet A.

[29] McGregor TL, Misri A, Bartlett J, Orabona G, Friedman RD, Sexton D (2010). Consanguinity mapping of congenital heart disease in a South Indian population. PLoS One.

[30] Girotto G, Mezzavilla M, Abdulhadi K, Vuckovic D, Vozzi D, Khalifa Alkowari M (2014). Consanguinity and hereditary hearing loss in Qatar. Hum Hered.

[31] Korf BR, Rehm HL (2013). New approaches to molecular diagnosis. JAMA.

[32] Aramesh K (2009). A closer look at the abortion debate in Iran. Am J Bioeth.

[33] Jafri H, Ahmed S, Ahmed M, Hewison J, Raashid Y, Sheridan E (2012). Islam and termination of pregnancy for genetic conditions in Pakistan: implications for Pakistani health care providers. Prenat Diagn.

[34] Alkuraya FS, Kilani RA (2001). Attitude of Saudi families affected with hemoglobinopathies towards prenatal screening and abortion and the influence of religious ruling (Fatwa). Prenat Diagn.

[35] Shoham-Vardi I, Weiner N, Weitzman D, Levcovich A (2004). Termination of pregnancy: attitudes and behavior of women in a traditional society. Prenat Diagn.

[36] Amin TT, Al-Wadaani HA, Al-Quaimi MM, Aldairi NA, Alkhateeb JM, Al-Jaafari AA (2012). Saudi women’s interest in breast cancer gene testing: possible influence of awareness, perceived risk and socio-demographic factors. Asian Pac J Cancer Prev.

[37] Al Sulaiman A, Saeedi M, Al Suliman A, Owaidah T (2010). Postmarital follow-up survey on high risk patients subjected to premarital screening program in Saudi Arabia. Prenat Diagn.

[38] Abotalib Z (2013). Preimplantation genetic diagnosis in Saudi Arabia. Bioinformation.

[39] Amagwula T, Chang PL, Hossain A, Tyner J, Rivers AL, Phelps JY (2012). Preimplantation genetic diagnosis: a systematic review of litigation in the face of new technology. Fertil Steril.

[40] Ayesh SK, Al-Sharef WA, Nassar SM, Thawabteh NA, Abu-Libdeh BY (2005). Prenatal diagnosis of beta-thalassemia in the West Bank and Gaza. Saudi Med J.

[41] Al Sulaiman A, Suliman A, Al Mishari M, Al Sawadi A, Owaidah TM (2008). Knowledge and attitude toward the hemoglobinopathies premarital screening program in Saudi Arabia: population-based survey. Hemoglobin.

[42] Gharaibeh H, Mater FK (2009). Young Syrian adults’ knowledge, perceptions and attitudes to premarital testing. Int Nurs Rev.

[43] Saniei M, Mehr EJ, Shahraz S, Zahedi LN, Rad AM, Sayar S (2008). Prenatal screening and counseling in Iran and ethical dilemmas. Community Genet.

[44] Belhoul KM, Abdulrahman M, Alraei RF (2013). Hemoglobinopathy carrier prevalence in the United Arab Emirates: first analysis of the Dubai Health Authority premarital screening program results. Hemoglobin.

[45] Biesecker LG (2010). Exome sequencing makes medical genomics a reality. Nat Genet.

[46] Veltman JA, Brunner HG (2012). De novo mutations in human genetic disease. Nat Rev Genet.

[47] Rehm HL (2013). Disease-targeted sequencing: a cornerstone in the clinic. Nat Rev Genet.

[48] Pierce BL, Ahsan H (2010). Clinical assessment incorporating a personal genome. Lancet.

[49] Alford RL, Arnos KS, Fox M, Lin JW, Palmer CG, Pandya A (2014). American college of medical genetics and genomics guideline for the clinical evaluation and etiologic diagnosis of hearing loss. Genet Med.

[50] Green RC, Berg JS, Grody WW, Kalia SS, Korf BR, Martin CL (2013). ACMG recommendations for reporting of incidental findings in clinical exome and genome sequencing. Genet Med.

